# Case report and literature review: IgG4-related tubulointerstitial nephritis coexistent with systemic lupus erythematosus

**DOI:** 10.3389/fmed.2025.1585351

**Published:** 2025-07-11

**Authors:** Xingfu Ji, Guilin Jing, Haoqi Sun, Xuan Li, Xuexun Chen

**Affiliations:** ^1^Department of Nephrology, Affiliated Hospital of Shandong Second Medical University, Weifang, China; ^2^Department of Nephrology, Sunshine Union Hospital, Weifang, China

**Keywords:** systemic lupus erythematosus, lupus nephritis, IgG-4 related diseases, IgG4-related tubulointerstitial nephritis, renal biopsy

## Abstract

We present the case of a 68-year-old man with acute kidney injury that occurred following radiofrequency cardiac ablation. Laboratory tests revealed elevated serum creatinine (SCr), positivity for antinuclear and anti-double-stranded DNA (anti-dsDNA) antibodies, elevated IgG4 levels, and hypocomplementemia. Renal biopsy identified coexisting mesangial proliferative lupus nephritis (LN) (Class II) and IgG4-related tubulointerstitial nephritis (IgG4-TIN). The patient responded well to glucocorticoid-based immunosuppressive therapy, with significant improvement in renal function. This case illustrates the rare coexistence of systemic lupus erythematosus (SLE) and IgG4-related kidney disease (IgG4-RKD), highlighting the importance of renal biopsy in distinguishing overlapping autoimmune nephropathies.

## Introduction

Chronic kidney disease is a major cause of morbidity and mortality globally, affecting more than 12% of the adult population (1, 2), which underscores the need to uncover molecular pathogenic mechanisms and treatment targets (3–5). Immunoglobulin (Ig)G4-related disease (IgG4-RD) involves inflammation and fibrosis of one or more organs and is characterized by three features: (1) IgG4-positive plasma cell infiltration; (2) varying degrees of fibrosis, typically displaying mat pattern fibers; and (3) increased blood IgG4 levels (6). IgG4-related kidney disease (IgG4-RKD), a subset of IgG4-RD, is divided into three categories: tubulointerstitial inflammation (IgG4-TIN), retroperitoneal fibrosis, and membranous nephropathy. IgG4-TIN is the most common of these categories.

Systemic lupus erythematosus (SLE) is an autoimmune disease characterized by a range of clinical manifestations. Immune disorders lead to the production of autoantibodies, affecting the normal functioning of many organs and systems. Abnormal antinuclear antibodies (ANAs), complement, and immunoglobulins form immune complexes and deposits in the glomeruli, leading to lupus nephritis (LN). LN, one of the most serious clinical manifestations of SLE, is characterized by proteinuria and progressive renal dysfunction (7).

Both IgG4-TIN and LN are autoimmune inflammatory diseases, but their coexistence is rare. Here, we report a case with simultaneous IgG4-TIN and LN.

## Case presentation

One month before admission to our hospital, a routine physical examination of a 68-year-old male patient revealed an abnormally elevated serum creatinine (SCr) level of 2.09 mg/dL (reference range: 0.67–1.18 mg/dL), despite the absence of other symptoms or signs of renal dysfunction. No further tests were performed at that time to determine the cause of the elevated SCr. Two weeks prior to admission, the patient experienced frequent premature ventricular contractions (PVCs) and palpitations, for which he underwent radiofrequency cardiac ablation using a small amount of contrast medium. Although SCr was 2.5 mg/dL during the post-procedural evaluation, no specific intervention was initiated, and the patient was advised to undergo regular follow-up. Five days before admission, the patient developed new-onset periorbital and bilateral lower extremity edema, accompanied by an increase in SCr to 5.74 mg/dL. Therefore, he was admitted to our hospital for treatment.

The patient had a history of PVCs, hypertension, benign prostatic hyperplasia, and gallstones. He was taking tamsulosin hydrochloride (0.2 mg, sustained-release tablet) once nightly, atorvastatin calcium (20 mg) once nightly, metoprolol succinate (47.5 mg, sustained-release) once daily, and nifedipine (30 mg, sustained-release) twice daily. Physical examination showed pallid conjunctiva, facial pallor, and edema of the eyelids and both lower limbs, and no other abnormal findings.

Laboratory tests revealed positivity for ANAs at a titer of 1:1000, elevated anti-double-stranded DNA (anti-dsDNA) antibodies at 197 IU/mL (reference: 0–120 IU/mL), and hypocomplementemia with reduced C3 (0.23 g/L; reference: 0.9–1.8 g/L) and C4 (0.02 g/L; reference: 0.1–0.4 g/L). Proteinuria was noted at 0.6 g/day (reference: 0–0.15 g/24 h), while antiphospholipid antibodies tested negative. Additionally, SCr was markedly elevated at 5.74 mg/dL (reference: 0.7–1.3 mg/dL), with serum IgG4 elevated to 1,180 mg/dL (reference: 3–201 mg/dL) and serum IgG to 2,030 mg/dL (reference: 700–1,600 mg/dL).

Chest computed tomography, cardiac ultrasound, and abdominal sonography showed liver cysts and gallstones, but no other abnormalities. Urological ultrasound showed preserved corticomedullary differentiation and mild separation of the right renal pelvis.

**Figure 1:**
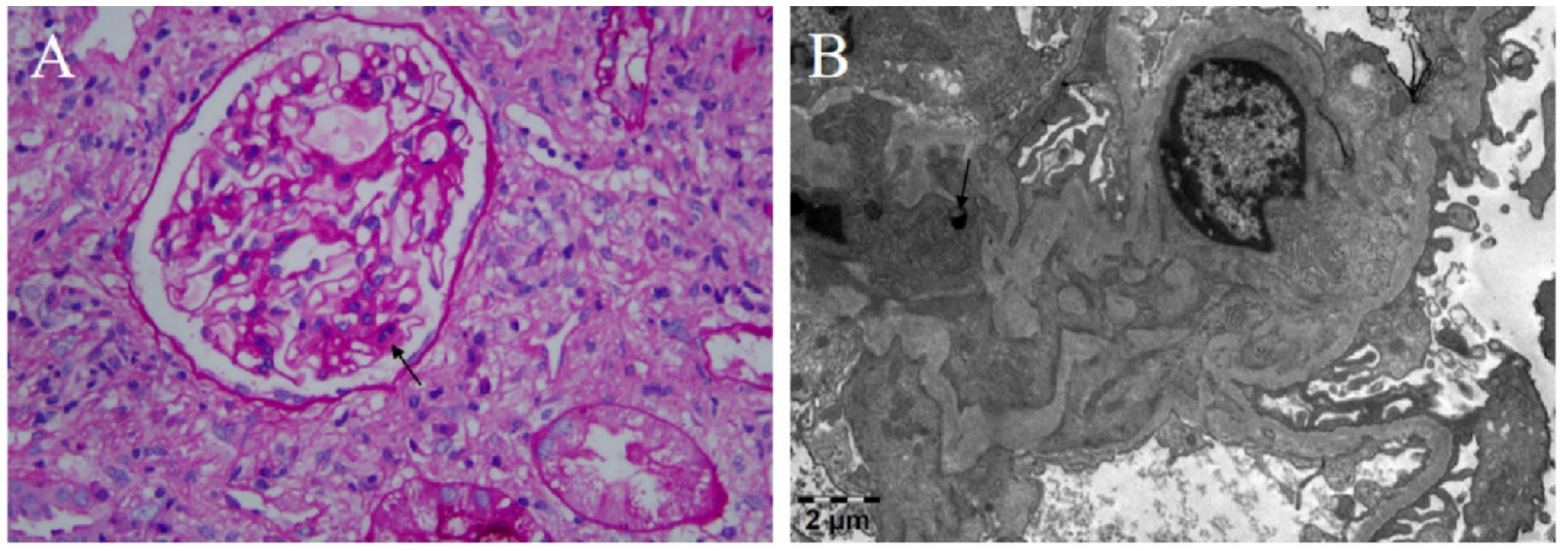
Renal biopsy findings. **(A)** Periodic Acid-Schiff staining shows mild mesangial cell and matrix proliferation (×400); arrow: mesangial hypercellularity. **(B)** Electron microscopy staining: electron-dense deposits (arrow) in the mesangial area (×1,900).

**Figure 2:**
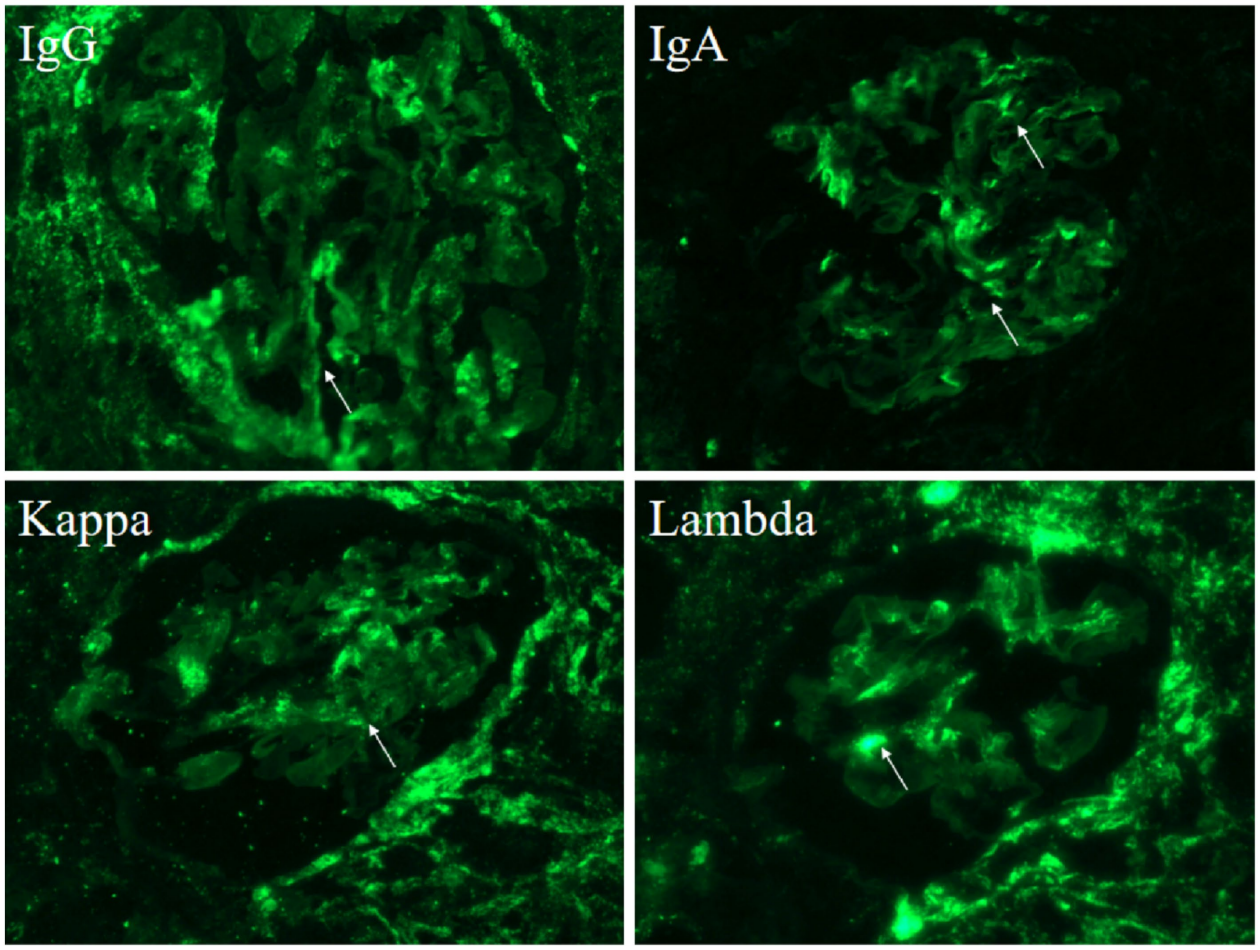
Immunofluorescence shows deposition of various immune complexes (arrow): IgG (++), IgA (++), Kappa (++), Lambda (++).

Renal biopsy findings under light microscopy showed mild mesangial hypercellularity (3–5 cells per mesangial area) and matrix expansion, with patent capillary loops and no evidence of basement membrane thickening, necrosis, or leukocyte infiltration (Figure 1A). Endothelial cells appeared unremarkable. Electron microscopy revealed prominent vacuolar changes in glomerular endothelial cells and mesangial matrix expansion with scattered electron-dense deposits in the mesangial areas (Figure 1B). Capillary lumina remained open. Immunofluorescence demonstrated granular mesangial deposition of immune complexes, including IgG (++), IgA (++), IgM (++), complement component C1q (+), Ig light chain Kappa (*κ*) (++), and Ig light chain Lambda (*λ*) (++) (Figure 2). These findings supported an immune complex-mediated injury pattern, consistent with the International Society of Nephrology (ISN)/Renal Pathology Society (RPS) Class II LN.

**Figure 3:**
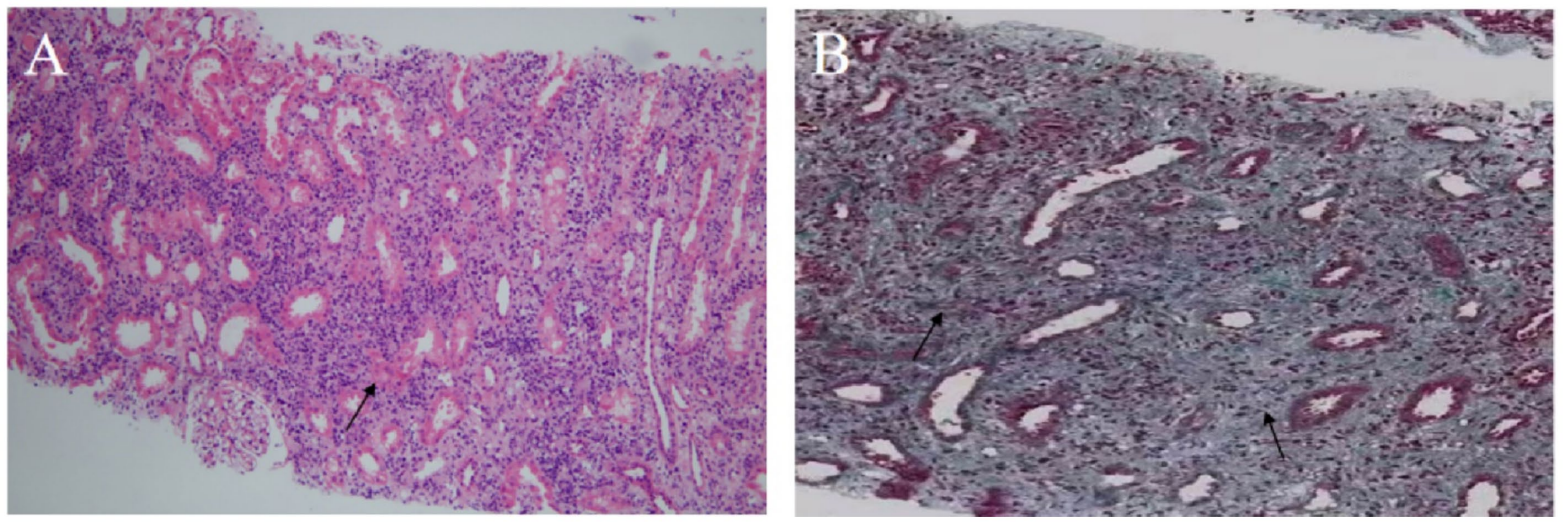
Renal biopsy findings. **(A)** Hematoxylin–eosin staining shows diffuse interstitial inflammatory infiltration with numerous plasma cells and tubular atrophy (×100); arrow: plasma cell infiltration. **(B)** Masson’s staining demonstrates storiform interstitial fibrosis (×200); arrow: area of storiform fibrosis.

**Figure 4:**
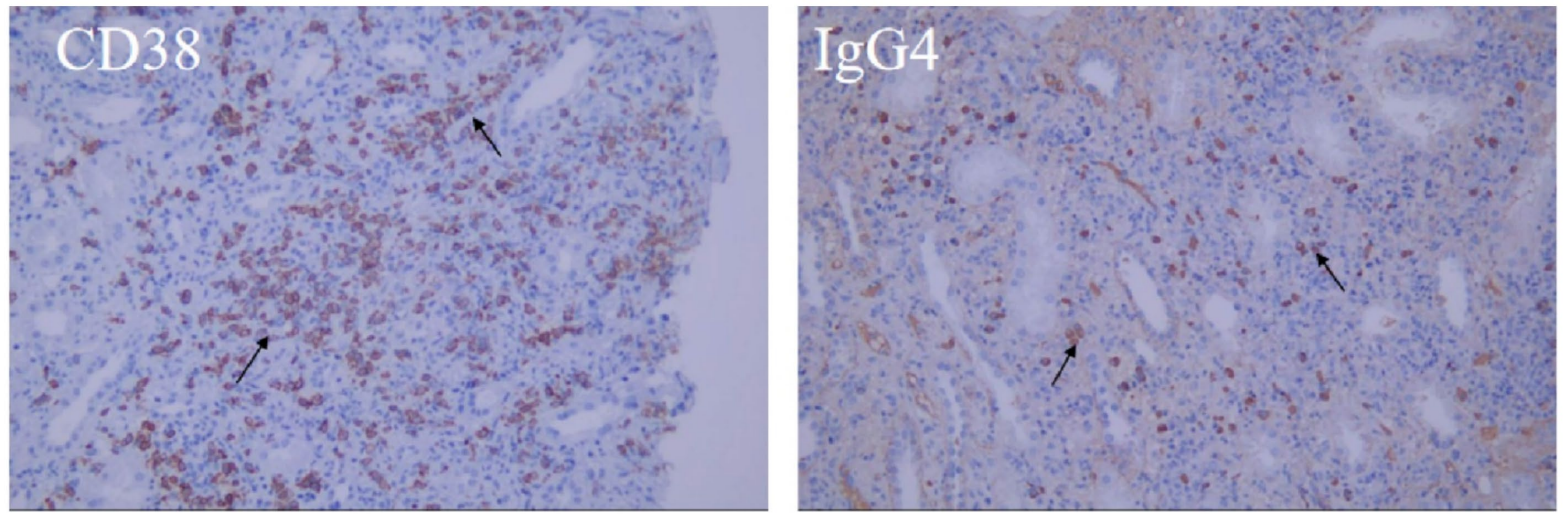
Immunohistochemistry: Multifocal cells of renal interstitium were positive for CD38, IgG4 (arrow).

Hematoxylin–eosin staining revealed diffuse interstitial inflammation rich in plasma cells and associated tubular injury (Figure 3A), while Masson’s trichrome staining showed prominent interstitial fibrosis with a storiform pattern (Figure 3B). Immunohistochemistry revealed an abundance of IgG4-positive plasma cells (greater than 30 per high-power field (/HPF)), with an IgG4/IgG-positive cell ratio greater than 40%, along with CD38 (Figure 4). These features indicated IgG4-RKD.

In summary, our patient presented with elevated ANA titers, increased anti-dsDNA antibodies, and hypocomplementemia, consistent with ISN/RPS Class II LN, as confirmed by renal biopsy. The 2019 European League Against Rheumatism (EULAR)/American College of Rheumatology (ACR) classification criteria yielded a total score of 18, fulfilling the diagnostic threshold for SLE (see Table 1) (8). Additionally, the patient exhibited elevated SCr and markedly increased IgG4 levels, with renal histopathology revealing storiform fibrosis, which was greater than 30 IgG4-positive plasma cells per high-power field, and the IgG4/IgG-positive cell ratio was greater than 40%. These findings met the 2011 diagnostic criteria for IgG4-RKD (items 1 + 3 + 4a + 4b), as defined by the Japanese Society of Nephrology (see Table 2) (9).

**Table 1:** Diagnostic criteria fulfilled by the patient: 2019 EULAR/ACR criteria for SLE.

Domain	Item	In this case	Score
Entry criterion	ANA	Present	positive
Clinical domains and criteria			
	Constitutional	Absent	-
	Fever		-
	Hematologic	Absent	
	Leukopenia		-
	Thrombocytopenia		-
	Autoimmune hemolysis		-
	Neuropsychiatric	Absent	-
	Delirium		-
	Psychosis		-
	Seizure		-
	Mucocutaneous	Absent	-
	Non-scarring alopecia		-
	Oral ulcers		-
	Subacute cutaneous or discoid lupus		-
	Acute cutaneous lupus		-
	Serosal	Absent	-
	Pleural or pericardial effusion		-
	Acute pericarditis		-
	Musculoskeletal	Absent	-
	Joint involvement		-
	Renal		-
	Proteinuria >0.5 g/24 h	Present	-
	Renal biopsy Class II or V lupus nephritis	Present	8
	Renal biopsy Class III or IV lupus nephritis	Absent	-
Immunology domains and criteria			
	Antiphospholipid antibodies		-
	Anti-cardiolipin antibodies OR anti-β2GP1 antibodies OR Lupus anticoagulant	Absent	-
	Complement proteins		-
	Low C3 OR low C4		-
	Low C3 AND low C4	Present	4
	SLE-specific antibodies		-
	Anti-dsDNA antibodies OR anti-Smith antibodies	Present	6
Total score	≥10: SLE classified		18

SLE diagnostic criteria: ANA >1: 80; total score ≥10 (2019 EULAR/ACR). SLE, systemic lupus erythematosus; ANA, antinuclear antibody; Anti-dsDNA, anti-double-stranded DNA antibody.

**Table 2:** Diagnostic criteria fulfilled by the patient: 2011 JSN criteria for IgG4-related kidney disease.

Diagnostic criteria for IgG4-related kidney disease (IgG4-RKD) 2011	Meets criteria
1. Presence of some kidney damage, as manifested by abnormal urinalysis or urine marker(s) or decreased kidney function with either elevated serum IgG level, hypocomplementemia, or elevated serum IgE level	Present
2. Abnormal renal radiologic findings:	Absent
Multiple low-density lesions on enhanced computed tomography	
Diffuse kidney enlargement	
Hypovascular solitary mass in the kidney	
Hypertrophic lesion of the renal pelvic wall without irregularity of the renal pelvic surface	
3. Elevated serum IgG4 level (IgG4 ≥ 135 mg/dL)	Present
4. Histologic findings in the kidney:	
Dense lymphoplasmacytic infiltration with infiltrating IgG4-positive plasma cells >10/high power field (HPF) and /or IgG4/IgG-positive plasma cells >40%	Present
Characteristic fibrosis surrounding nests of lymphocytes and/or plasma cells	Present
5. Histologic findings in extra-renal organ(s): Dense lymphoplasmacytic infiltration with infiltrating IgG4-positive plasma cells >10/HPF and/or IgG4/IgG-positive plasma cells >40% in extra-renal organ(s) Imaging or clinical findings in extra-renal organ(s): existence of one of the following items: Bilateral lacrimal gland swelling Bilateral submandibular or parotid gland swelling Imaging findings compatible with type 1 autoimmune pancreatitis Imaging features of retroperitoneal fibrosis	Absent
This case fulfills items	1 + 3 + 4a + 4b

IgG4-RKD definite diagnostic criteria: 1 + 3 + 4a + 4b, or 2 + 3 + 4a + 4b, or 2 + 3 + 5a, or 1 + 3 + 4a + 5a or 5b, or 2 + 3 + 4a + 5b.

Immunosuppressive therapy was initiated based on the patient’s SLE Disease Activity Index (SLEDAI) score of 12 points, in accordance with the 2023 EULAR recommendations for LN and the international consensus on IgG4-RKD (10–12). A personalized treatment strategy was adopted, starting with intravenous methylprednisolone (40 mg/day) combined with low-dose cyclophosphamide (CTX, 0.2 g per dose × 3) to induce remission. In October 2022, the patient’s SCr declined to 1.69 mg/dL, anti-dsDNA antibody titer decreased to 53 IU/mL, ANAs became negative, and the SLEDAI score dropped to 8. Prednisone was reduced to 20 mg/day, and the cumulative CTX dose reached 1.8 g. By January 2023, the patient’s renal function normalized (SCr: 1.18 mg/dL), and mycophenolate mofetil (MMF; 0.75 g twice daily) was introduced while prednisone was tapered to 5 mg/day. At the recent follow-up in January 2025, SCr had further improved to 0.85 mg/dL, with persistently negative ANAs, normalized complement levels (C3: 0.74 g/L; C4: 0.20 g/L), a low anti-dsDNA antibody titer (49 IU/mL), and a SLEDAI score of 0, indicating sustained disease remission. The key follow-up indicators and treatment course of the patient are summarized in Table 3.

**Table 3:** Key follow-up indicators and treatment timeline of the patient.

Date	SCr (mg/dL)	eGFR* (ml/min × 1.73 m^2^)	ANA	A-dsDNA (IU/mL)	C3 (g/L)	C4 (g/L)	SLE DAI	Treatment
July 2022	5.74	13.11	Positive	197	0.23	0.02	12	Methylprednisolone 40 mg/day (IV) + CTX (IV) 0.2 g per dose × 3
Sep 2022	2.37	26.01	Positive	88	0.69	0.19	10	Oral prednisone 40 mg/day tapering; CTX (IV) 0.2 g per dose × 3
Oct 2022	1.69	37.42	Negative	53	0.63	0.19	8	Prednisone 20 mg/day; CTX (cumulative 1.8 g)
Jan 2023	1.18	49.65	-	-	-	-	-	Prednisone 5 mg/day +MMF started (0.75 g BID)
Apr 2023	1.1	56.96	-	-	-	-	-	MMF maintenance
July 2023	0.88	79.57	Negative	91	0.73	0.17	4	MMF tapering phase
Mar 2024	0.99	76.75	Negative	69.1	0.82	0.19	0	MMF withdrawal
Jan 2025	0.85	87.65	Negative	49	0.74	0.20	0	Prednisone 5 mg/day only

*eGFR was calculated using the CKD-EPI equation (Chronic Kidney Disease Epidemiology Collaboration) (32); ANA, antinuclear antibody; A-dsDNA, anti-double-stranded DNA antibody; SCr, serum creatinine; SLE DAI, Systemic Lupus Erythematosus Disease Activity Index; MMF, mycophenolate mofetil; CTX, cyclophosphamide; IV, intravenous injection; −, not checked.

## Discussion

Previous kidney biopsy analyses showed IgG4-TIN with concurrent glomerular injury-associated kidney disease (13, 14). Here, we present a rare case of the simultaneous diagnosis of SLE with IgG4-RKD, highlighting two distinct autoimmune diseases with overlapping renal manifestations. Although both conditions can involve the kidneys, their coexistence within the same individual is exceedingly rare, posing unique diagnostic and therapeutic challenges.

Our case patient experienced frequent PVCs. Although his antiphospholipid antibodies were negative, PVCs were reported during the active phases of SLE, which was likely attributed to autoimmune-mediated myocardial or conduction system involvement (15). Thus, an SLE-related arrhythmia could not be excluded. Additionally, there are currently no documented cases of IgG4-RD presenting with cardiac arrhythmias, making IgG4-RD an unlikely contributor in this context. During arrhythmia management, the patient underwent radiofrequency catheter ablation, requiring the administration of iodinated contrast media. Given the elevated SCr, contrast-induced nephropathy (CIN) was initially suspected. However, renal biopsy showed predominant plasma cell infiltration and storiform interstitial fibrosis, without eosinophilic infiltration or interstitial edema typical of CIN. These histopathologic features ruled out CIN in this patient.

Systemic lupus erythematosus is a multisystem autoimmune disease with strong female predominance, typically affecting women aged 20–40 years. LN is a common and serious complication of SLE, affecting approximately 14–55% of patients, with prevalence varying based on ethnicity and geographic region (16). The 2018 revised ISN/RPS classification stratifies LN based on immune complex deposition patterns, with Class II characterized by mesangial hypercellularity and immune deposits without significant glomerular basement membrane involvement (17). Serologically, positive anti-dsDNA antibodies and low complement (C3, C4) levels reflect disease activity.

IgG4-RD is a systemic, immune-mediated fibroinflammatory condition characterized by organ enlargement, elevated serum IgG4, and tissue infiltration by IgG4-positive plasma cells. In the kidney, the predominant manifestation is IgG4-TIN, typically observed in middle-aged and elderly men (9).

Despite overlapping renal manifestations, SLE and IgG4-RKD represent distinct immunologic entities. Histologically, LN typically affects glomeruli, whereas IgG4-RKD predominantly involves the tubulointerstitial compartment. Our patient simultaneously met the diagnostic criteria for both SLE (EULAR/ACR 2019) and IgG4-RKD (Japanese Society of Nephrology 2011) (8, 9). The clinical significance of differentiating SLE from IgG4-RKD lies in their distinct therapeutic strategies and prognostic implications. Although both conditions can affect the kidneys, misclassification could lead to overtreatment or undertreatment. For instance, unnecessary exposure to cytotoxic immunosuppressants can be avoided in patients correctly diagnosed with IgG4-TIN, which often responds well to corticosteroids alone. Conversely, failure to recognize LN can delay the timely initiation of disease-modifying therapies, increasing the risk of irreversible renal damage. Therefore, establishing an accurate diagnosis is essential for tailoring appropriate treatment and optimizing patient outcomes.

To better contextualize our case, we reviewed five published reports of patients diagnosed with both SLE and IgG4-RD (18–22), which are summarized in Table 4. This revealed a consistent pattern of elderly male predominance, mixed systemic and renal symptoms, and dual serologic and histopathologic features. All patients exhibited positivity for ANAs and anti-dsDNA antibodies, while complement levels varied. Renal biopsy findings ranged from lupus-associated glomerulonephritis to IgG4-TIN, often coexisting within the same specimen. Despite pathologic heterogeneity, the presence of abundant IgG4^+^ plasma cells (> 10–40/high-power field) (18, 20–22), storiform fibrosis (21, 22), and an IgG4/IgG-positive cell ratio > 40% (19, 20) in several of these cases supports true IgG4-RKD rather than incidental IgG4 elevation. All patients received glucocorticoid-based immunosuppressive therapy, with or without additional agents (MMF, HCQ, belimumab), and experienced clinical improvement. These cases, along with our case patient, highlight the diagnostic value of IgG subclass staining in lupus patients with atypical interstitial pathology. Standard SLE-directed therapy appears effective in such overlap syndromes, but the pathogenic role and prognostic relevance of IgG4 components warrant further investigation. Increasing evidence suggests that the molecular mechanisms underlying IgG4-TIN may be associated with retroperitoneal fibrosis, activation of the renin–angiotensin system, and microbial dysbiosis (23–25).

**Table 4:** Clinical data of 5 SLE patients complicated with IgG4-RD.

Author (Year)	Age/Sex	IgG4 (mg/dl)	Main clinical symptoms	Test Results	Pathology	Treatment plan	Therapeutic effect
Zaarour et al. (18)	71/ female	37.10	Abdominal pain, vomiting, and diarrhea; AKI with hematuria and proteinuria	ANA positive; hypoalbuminemia; creatinine 9.56 mg/dL, BUN 88 mg/dL	Kidney: Acute tubular necrosis, with a maximum of 13 IgG4 + cells /HPF. “full-house” of immune reactants.	GC + MMF	Showed improvement
Arai et al. (19)	74/male	1,210	Raynaud’s, arthritis, parotid swelling, and lymphadenopathy; later fever, dyspnea, and pleural effusion	ANA positive, low complement, elevated globulin, Anti-dsDNA positive	Kidney: Significant IgG4-positive infiltration in tubulointerstitium, IgG4+/ IgG + >40%	GC	Showed improvement
Yamamoto et al. (20)	58/ female	1,240	Photosensitivity and extremity rash; 2016: fever and fatigue.	ANA and anti-dSDNA are positive, normal complement, elevated IgG, and massive proteinuria	Kidney: Abundant IgG4-positive cells in the renal tubulointerstitium	GC + MMF; GC + Belimumab	Showed improvement
Ying et al. (22)	64/male	2,960	Recurrent lower limb erythema for 3 months	Elevated SCr, ANA and Anti-dsDNA positive, hemolytic anemia, 0.5 g/d proteinuria; CT: multiple lymphadenopathies	IgG4-related tubulointerstitial nephritis; mesangial proliferative glomerulonephritis with immune complex deposits	GC	Showed improvement
Xie et al. (21)	67/male	1700	Loss of appetite and general weakness	Leukopenia, thrombocytopenia; ANA and Anti-dsDNA positive; low C3/C4; elevated IgG	Multifocal lymphoid hyperplasia and interstitial fibrosis; >10 IgG4 + cells/HPF	GC + HCQ + MMF	Showed improvement
This case	68/male	1,180	periorbital and bilateral lower extremity edema	Elevated SCr, ANA and Anti-dsDNA positive; low C3/C4; and elevated IgG4	“full-house” of immune reactants, >30 IgG4 + cells /HPF; IgG4+/IgG + >40%	GC + CTX; GC + MMF	Showed improvement

GC, Glucocorticoid; HCQ, Hydroxychloroquine; MMF, Mycophenolate mofetil; HPF, high-power visual field; CTX, Cyclophosphamide.

Although the overlapping pathophysiology of IgG4-RD and SLE remains unclear, dysregulation of the humoral immune system is a common feature of the two diseases. In IgG4-RD, the expansion of CD20^+^ B cells and plasmablasts, as well as antigen presentation to CD4^+^ cytotoxic T cells, have been implicated in disease pathogenesis (26–28). Notably, T follicular helper (Tfh) cells facilitate B-cell differentiation into plasmablasts and contribute to IgG4 class-switching, suggesting a pivotal role in IgG4-RD progression (29). In SLE, which is also an autoimmune disease, impaired humoral regulation and hyperactivation of CD4^+^ T cells are prominent features. Tfh cells, which are significantly increased in patients with SLE, are known to promote autoreactive B-cell responses and autoantibody production, thereby driving disease progression (30, 31). These findings suggest that Tfh cells may act as a shared immunological hub linking IgG4-RD and SLE. Whether the dysregulation of the Tfh cell axis constitutes a common pathogenic mechanism in the two diseases remains to be further elucidated (32). Investigating this potential convergence may provide deeper insights into their overlapping features and guide targeted immunomodulatory therapies.

Our case patient was diagnosed with overlapping Class II LN and IgG4-RKD, presenting with acute kidney injury, hypocomplementemia, and elevated disease activity. Although Class II LN typically requires limited immunosuppression, the presence of renal dysfunction (SCr 5.74 mg/dL; estimated glomerular filtration rate [eGFR] 13.1 mL/min) warranted an intensified approach. Following the 2023 EULAR recommendations for SLE and the 2015 international consensus on IgG4-RD management, we induced remission by initiating intravenous methylprednisolone and low-dose CTX (11, 12). As renal function improved, glucocorticoids were tapered, and MMF was introduced as a steroid-sparing agent for long-term maintenance. The patient achieved sustained clinical remission, allowing MMF tapering and maintenance with low-dose prednisone alone. This stepwise immunosuppressive strategy of balancing early disease control with long-term safety proved effective in managing both the LN and the IgG4-RKD.

## Conclusion

In this study, we report a rare case of overlapping LN and IgG4-TIN that highlights the importance of distinguishing CIN from immune-mediated renal dysfunction. This case shows that SLE and IgG4-TIN can coexist, though reports remain scarce. Our treatment strategy involves glucocorticoids combined with CTX proved effective, offering valuable therapeutic insights into managing complex presentations of SLE with IgG4-TIN.

## Data Availability

The original contributions presented in the study are included in the article/supplementary material, further inquiries can be directed to the corresponding authors.
